# Respiratory viral infection in early life and development of asthma in childhood: A protocol for systematic review and meta-analysis

**DOI:** 10.1097/MD.0000000000015419

**Published:** 2019-05-03

**Authors:** Md Zakiul Hassan, Muhammad Abdul Baker Chowdhury, Imran Hassan, Fahmida Chowdhury, Nancy Schaefer, Mohammod Jobayer Chisti

**Affiliations:** a Respiratory Infections Research Group, Programme for Emerging Infections, Infectious Diseases Division, icddr,b, Dhaka, Bangladesh; b Department of Emergency Medicine, University of Florida, Gainesville, Florida; c Laboratory Sciences and Services Division, icddr,b, Dhaka, Bangladesh; d Health Science Center Libraries, University of Florida, Gainesville, Florida; e Nutrition and Clinical Services Division, icddr,b, Dhaka, Bangladesh.

**Keywords:** acute respiratory infections, allergic sensitization, childhood asthma, childhood viral infections, recurrent wheezing, respiratory syncytial virus, rhinovirus

## Abstract

**Background:**

Respiratory viruses are the leading cause of early life wheezing that may contribute to the development of childhood asthma leading to increasing morbidity and socioeconomic burden. The aim of this review is to identify whether respiratory viral infections during first year of life were associated with development of childhood asthma.

**Methods:**

We will search major scientific databases (MEDLINE, CINAHL, Web of Science, Cochrane Library, and ClinicalTrials.gov) using truncated and phrase-searched keywords and relevant subject headings. Observational studies including case–control studies, cohort studies, and randomized control trails published in English will be included in this review. Case reports, qualitative studies, and narrative overviews will be excluded. Exposure will be defined as laboratory-confirmed viral respiratory tract infection in the first year of life and outcome will be defined as development of asthma between ages 5 and 18 years. Effect sizes in bivariate and multivariate analyses will be presented as odds or prevalence ratios. We will explore for heterogeneity of the standard errors across the studies, and if appropriate, we will perform a meta-analysis using a random-effects model to present a summary estimate of the odds or prevalence ratios.

**Results:**

This review will assess whether respiratory viral infections during first year of life increases the risk of childhood asthma development.

**Conclusions:**

This systematic review will evaluate published literature, assessing the link between early life viral infection and childhood asthma. Pooled data may provide evidence that infantile respiratory viral infection results in higher incidence of recurrent wheezing and asthma, thereby stimulating further research into the cost-effectiveness of pharmaceutical interventions such as vaccines and nonpharmaceutical interventions such as hand-washing and respiratory hygiene promotion to young children. Implementing the results of such research may then reduce the burden of acute viral respiratory infections and subsequent recurrent wheezing and asthma.

**Systematic review registration:**

This systematic review has been registered on PROSPERO (CRD42018105519)

## Abstract 2

Supplemental Digital Content is available in the text

## 1 Introduction

Respiratory viral wheezing illness is common in early childhood.^[1,2]^ Frequently identified respiratory viruses associated with these wheezing episodes include respiratory syncytial virus (RSV), human rhinovirus (RV), metapneumovirus, influenza virus, parainfluenza virus, and adenovirus.^[3–5]^ Several epidemiological studies reported that respiratory virus associated wheezing during early life, particularly during infancy, contributes to the development of asthma during childhood.^[6–8]^ Studies have also investigated the etiologic role of specific viruses in the development of asthma.^[9,10]^ The Childhood Origins of ASThma (COAST) birth cohort found that wheezing illnesses with RV in the first 3 years of life were the most significant factor for subsequent asthma development in high-risk children at 6 years of age, regardless of aeroallergen sensitization or other risk factors.^[11]^ Similarly, children hospitalized for RSV bronchiolitis during first 12 months of life were 2.5 times more likely to develop subsequent asthma at age of 91 months than children without RSV-bronchiolitis.^[12]^ A birth cohort study has also demonstrated the high prevalence of asthma following severe RSV bronchiolitis, with 21% of infants hospitalized for RSV bronchiolitis having asthma at the age of 6 years compared with 5% of the control cohort.^[13]^

However, many previous studies have documented contradictory results reporting that early life infections protect from allergic diseases including childhood asthma (hygiene hypothesis).^[14–16]^ In addition, recent evidence have also suggested that the number of respiratory episodes in the first years of life rather than specific viral trigger was associated with subsequent childhood asthma.^[17]^ This discrepancy in the literature is important to address, as it may affect how risk for childhood asthma is ascertained; accurate identification of high-risk children may be key in asthma prevention.

Asthma is the most common chronic disease in childhood, placing a great burden on children, their families, and society.^[18–20]^ Available treatments reduce morbidity during treatment but do not alter the natural history of the disorders.^[21]^ Research related to the risk and protective factors is thus a priority for public health.

## 2 Aim and review question

The overall aim of this systematic review is to identify whether respiratory viral infections during first year of life were associated with development of childhood asthma. In this review, we aim to understand whether respiratory viral infection during first year of life increases the risk of subsequent asthma development between age 5 and 18 years.

## 3 Methods

### 3.1 Study design

This systematic review protocol will be reported in accordance with the Preferred Reporting Items for Systematic Review and Meta-Analyses Protocols (PRISMA-P) 2015 Statement.^[22]^ A populated checklist for this review protocol has been provided as PRISMA-P checklist (Additional file-1). The protocol has been registered in PROSPERO (CRD42018105519).

### 3.2 Eligibility criteria

The study will include all research studies with available full texts published since inception of the selected databases to June 30, 2018. All articles published in English on human subject research will be considered. This study will include both interventional and observational studies, inclusive of randomized controlled trials, cohort studies as well as case–control studies. Case reports, qualitative studies, and narrative overviews will be excluded.

### 3.3 Information sources

We will search the major databases [MEDLINE (PubMed), CINAHL, Web of Science, the Cochrane Library, and ClinicalTrials.gov] using truncated and phrase-searched keywords and relevant subject headings based on the 5 concepts of triggering condition, age, risk/cause/history, and resulting conditions. For unclear or unreported parameters in the studies such as the number of participants, efforts will be made to contact the authors and obtain the missing information.

### 3.4 Search strategy

**Figure 1:**
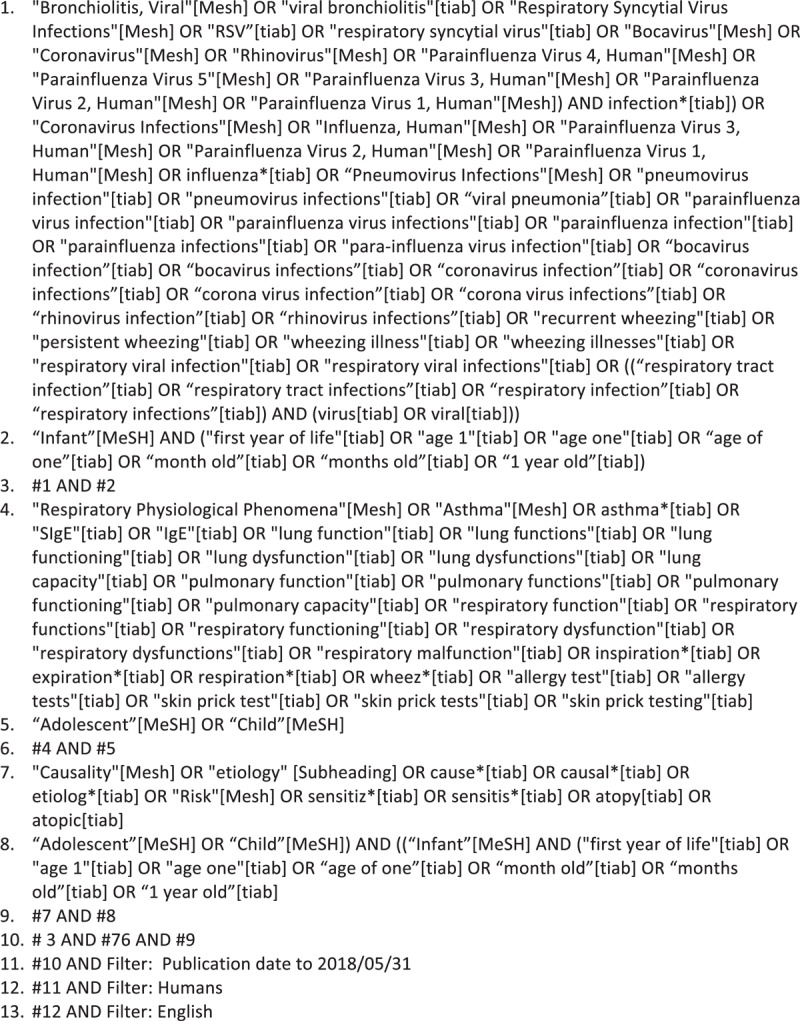
PubMed tentative search strategy.

A draft literature search strategy for MEDLINE database has been developed by an experienced librarian and systematic reviewer (NS) in consultation with other authors using a combination of Medical Subject Headings (MeSH), keyword terms, and filters. The complete search strategy for MEDLINE database is presented in Fig. 1. These search terms will be adapted for each of the databases to take into account differences in controlled vocabulary and syntax rules.

### 3.5 Condition/domain being studied

The conditions being studied included asthma, respiratory infections, and lung function.

### 3.6 Population/participants

We are interested in studies on participants aged 0 to 18 years with data on laboratory-confirmed respiratory viral infections during first year of life and with information on recurrent wheezing/asthma/lung function.

### 3.7 Exposure

Respiratory viral infections in the first year of life.

### 3.8 Comparator (s)/control

The study group will be children who may have had at least 1 episode of specific respiratory viral infection during their first year of life. The control group can include children without any specific respiratory viral infection during first year of life.

### 3.9 Context

All countries and all settings will be included.

### 3.10 Outcome

Our primary outcome would be asthma/recurrent wheezing/physician-diagnosed asthma/asthma medication use/parental-reported asthma/lung function.

#### 3.10.1 Asthma

Three or more episodes of physician-verified wheezing in a year.

#### 3.10.2 Recurrent wheezing

Three or more episodes, reported by a parent but not verified by a physician.

#### 3.10.3 Physician-diagnosed asthma

An illness given the following specific diagnoses by a registered physician during hospital visit: bronchiolitis, wheezing illness, postinfective wheeze, reactive airway disease, asthma, or asthma exacerbation OR a positive response to the following question “Has a doctor ever actually said that your child has asthma”?

#### 3.10.4 Asthma medication use

An illness for which the child was prescribed short-acting or long-acting Beta-agonists and/or long-term controller medications.

#### 3.10.5 Parenteral-reported asthma/wheezing

Parenteral-reported asthma/wheezing was reported by parents when asked whether the child had ever wheezed during the past year.

#### 3.10.6 Lung function

FEV1 (forced expiratory volume in 1 second), forced vital capacity (FVC), and the FEV1/FVC ratio.

### 3.11 Data extraction (selection and coding)

Two independent reviewers will screen the titles and/or abstracts of studies retrieved and will remove studies that fail to meet the inclusion criteria mentioned above. The 2 reviewers will independently assess full texts of eligible studies and will also assess study quality. Disagreement over the eligibility of particular studies will be resolved through discussion with a third author.

We will use a pre-tested structured form to extract data from the included studies and evidence synthesis. Extracted information will include study setting, study population and participant demographics and baseline characteristics, details of exposure measurement, study methodology, recruitment and study completion rates, outcomes, and interval between exposure and outcome measurements information for assessment of the risk of bias. Two review authors will extract data independently; discrepancies will be identified and resolved through discussion with a third author where necessary. Missing data will be requested from study authors.

### 3.12 Risk of bias (quality) assessment

We will use Cochrane risk of bias tools (ROBINS-1 and RoB 2.0) to assess study quality.^[23]^ Two review authors will independently assess the risk of bias in studies being considered after full-text review. Disagreements between the review authors over the risk of bias in particular studies will be resolved by discussion, with involvement of a third review author where necessary.

### 3.13 Strategy for data synthesis

We will pool the relative risk and odds ratio estimates using the inverse of variance as a weight. When heterogeneity of effects is detected, we will use the random effects model.

### 3.14 Analysis of subgroups or subsets

If the necessary data are available, subgroup analyses will be performed for children by age, type of infection, gender, and family history of allergy or asthma.

### 3.15 Dissemination plans

A manuscript will be developed and submitted for publication.

## 4 Discussion

Childhood asthma is common and has substantial impact on quality of life.^[24]^ Understanding modifiable early life risk exposures such as viral infections for the development of childhood asthma could be useful for designing and testing early life interventions. The outcomes of this systematic review will further our knowledge on the natural history of childhood wheezing and asthma. The results of this review may lead to future research on various low-cost preventive strategies. Pharmaceutical interventions such as vaccine and nonpharmaceutical interventions such as promotion of hand washing and respiratory hygiene among young children to reduce the burden of early life acute viral respiratory infections can be tested for cost-effectiveness. Implementation of such interventions apart from reducing the burden of acute respiratory illness could also reduce the burden of recurrent wheezing and asthma among children.

## Acknowledgments

MZH, FC, MIH, and MJC acknowledge the salary support from icddr,b, through a grant from the US Centers for Disease Control and Prevention.

## Author contributions

MZH conceived the idea and designed the study. MZH and MABC drafted the protocol, MIH revised the protocol, and FC and MJC critically reviewed it. NS refined the search parameters in consultation with the other authors, performed the searches, and removed duplicate citations. All authors read and approved the final protocol manuscript.

**Conceptualization:** Md. Zakiul Hassan.

**Formal analysis:** Md. Zakiul Hassan, Muhammad Abdul Baker Chowdhury, Md Imran Hassan, Fahmida Chowdhury, Nancy Schaefer, Mohammod Jobayer Chisti.

**Methodology:** Md. Zakiul Hassan, Muhammad Abdul Baker Chowdhury, Md Imran Hassan, Fahmida Chowdhury, Nancy Schaefer, Mohammod Jobayer Chisti.

**Software:** Nancy Schaefer.

**Supervision:** Muhammad Abdul Baker Chowdhury, Fahmida Chowdhury, Mohammod Jobayer Chisti.

**Validation:** Muhammad Abdul Baker Chowdhury, Nancy Schaefer.

**Writing – original draft:** Md. Zakiul Hassan.

**Writing – review & editing:** Muhammad Abdul Baker Chowdhury, Md Imran Hassan, Fahmida Chowdhury, Nancy Schaefer, Mohammod Jobayer Chisti.

Md. Zakiul Hassan orcid: 0000-0001-5888-1558.

## Supplementary Material

Supplemental Digital Content
