# Respiratory viral infection in early life and development of asthma in childhood: A protocol for systematic review and meta-analysis: Erratum

**DOI:** 10.1097/MD.0000000000015918

**Published:** 2019-05-31

In the article, “Respiratory viral infection in early life and development of asthma in childhood: A protocol for systematic review and meta-analysis”,^[1]^ which appears in Volume 98, Issue 18 of *Medicine*, Md Imran Hasan's name appears incorrectly as Imran Hasan.

The second sentence of the Methods sentence of the abstract has trials misspelled as trails and should be written as “Observational studies including case–control studies, cohort studies, and randomized control trials published in English will be included in this review.”

Point 10 of Figure 1 should be #3 AND #6 AND #9 instead of #3 AND #76 AND #9.
